# Tetrandrine is a potent cell autophagy agonist via activated intracellular reactive oxygen species

**DOI:** 10.1186/2045-3701-5-4

**Published:** 2015-01-14

**Authors:** Haiqing Wang, Ting Liu, Lu Li, Qin Wang, Chunrong Yu, Xin Liu, Wenhua Li

**Affiliations:** College of Life Sciences, Wuhan University, Wuhan, 430072 P R China; Ministry of Education Laboratory of Combinatorial Biosynthesis and Drug Discovery, College of pharmacy, Wuhan University, Wuhan, 430072 P R China

**Keywords:** Tetrandrine, Autophagy, Reactive oxygen species

## Abstract

**Background:**

Autophagy is an evolutionarily conserved cellular process that involves the lysosomal degradation of proteins and organelles and the recycling of cellular components to ensure cellular survival under external or internal stress. Numerous data has indicated that autophagy can be successfully targeted for the treatment of multiple cancers. We have previously demonstrated that tetrandrine, a bisbenzylisoquinoline alkaloid isolated from the broadly used Chinese medicinal herb *Stephaniae tetrandrae*, exhibits potent antitumor effects when used either alone or in combination with other drugs.

**Results:**

In the present study, we showed that tetrandrine is a broad-spectrum potent autophagy agonist. Although low-dose tetrandrine treatment does not affect cell viability, it can potently induce autophagy in a variety of cell lines, including cancerous cells and nontumorigenic cells. The autophagy inhibitors 3-methyladenine (3-MA) and chloroquine (CQ), effectively blocked tetrandrine-induced autophagy. Moreover, tetrandrine significantly triggered the induction of mitophagy. The underlying mechanisms are associated with the tetrandrine-induced production of intracellular reactive oxygen species (ROS), which plays a critical role in tetrandrine-induced autophagy.

**Conclusions:**

Here, we report that tetrandrine is a potent cell autophagy agonist and may have a wide range of applications in the fields of antitumor therapy and basic scientific research.

## Background

Three types of autophagy have been characterized: macroautophagy, microautophagy, and chaperone-mediated autophagy [1]. Macroautophagy (usually referred to simply as autophagy) is an evolutionarily conserved cellular process that involves the lysosomal degradation of proteins, organelles and other cellular components and the recycling of cellular components to ensure cellular survival when cells experience starvation or other stimuli [2]. Autophagy serves as a temporary survival mechanism that plays crucial roles in maintaining intercellular homeostasis, remodeling development, and regulating metabolism and the immune response, and is also associated with various human diseases and diverse stresses [3–5]. Ionizing radiation and diverse classes of anticancer agents usually affect autophagy, causing high levels of autophagosome accumulation and/or increasing autophagic flux [6]. The reported effects of autophagy on cancer therapy appear to be contradictory: while many studies have suggested that autophagy induction is a mechanism of chemoresistance, other investigations have concluded that autophagy is actually necessary for the antitumor effect of drugs [7–10]. For many drugs, though the role and the molecular mechanisms that underlie the effects on autophagy are still unclear, they have been widely used in clinical treatment or clinical trials [6, 11]. In this regard, both potent autophagy agonists and autophagy inhibitors may exhibit potential in clinical treatment [12, 13].

Tetrandrine is one member of the bisbenzylisoquinoline alkaloids isolated from the root of a traditional Chinese medicinal herb, *Stephaniae tetrandrae*, which has been broadly applied in clinical treatment for thousands of years in China [14]. In recent decades, it has been used to treat patients with rheumatoid arthritis [15], hypertension [16], sepsis [17], inflammation [18, 19], occlusive cardiovascular disorders [20] and silicosis [21] in modern medicine [22–24]. Due to its action on intracellular multiple signaling molecules and relatively low toxicity to humans even when administered at high doses, tetrandrine has been attracted considerable attention as an antitumor therapeutic [25–28]. We have previously demonstrated that tetrandrine induces apoptosis at high concentrations and stimulates autophagy at low concentrations in human HCC cells, and shows synergistic antitumour effects in combination with other chemotherapy agents [29–31].

In this study, we found that tetrandrine is a broad-spectrum potent autophagy agonist with effects on a variety of cell lines, including cancerous cells and nontumorigenic cells. Tetrandrine exhibits a much stronger activity in inducing autophagy than rapamycin. Moreover, our data show that the accumulation of intracellular reactive oxygen species (ROS) plays a critical role in tetrandrine-induced autophagy.

## Results

### Low-dose tetrandrine does not affect cell viability

**Figure 1:**
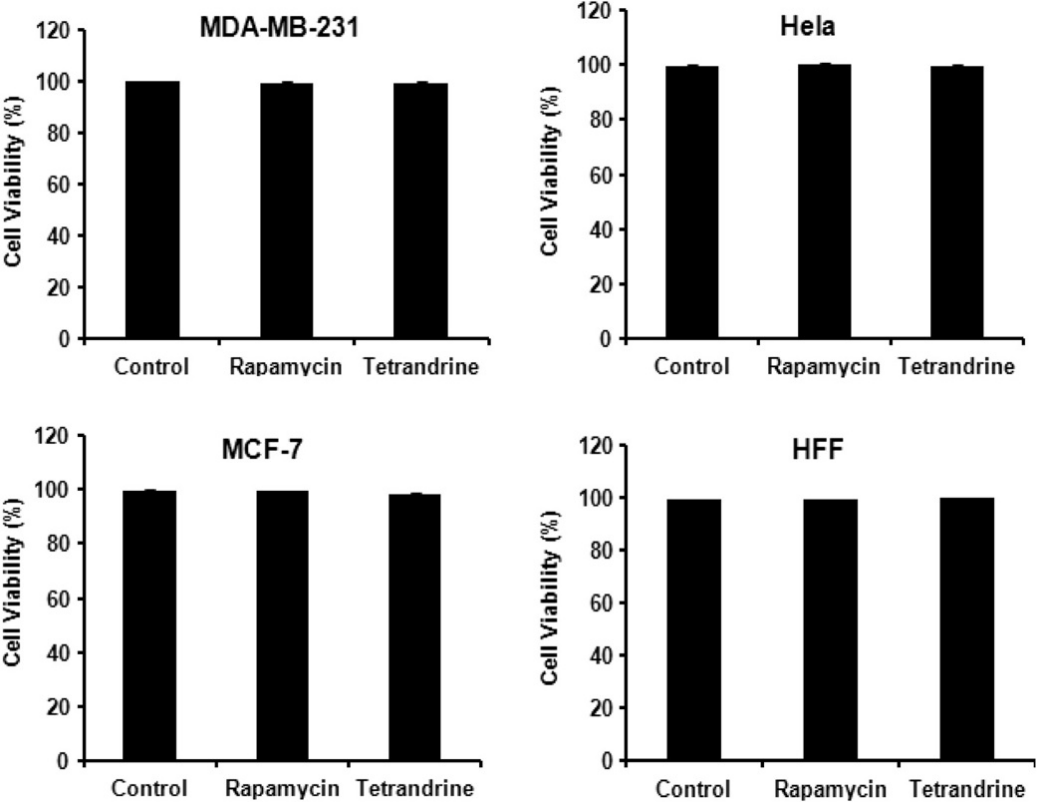
**Low-dose tetrandrine does not affect cell viability.** Data are representative of values from at least three independent experiments. The MDA-MB-231, MCF-7, and Hela cancer cells, as well as immortalized nonmalignant cells HFF, were treated with rapamycin or tetrandrine alone for 24 hours. Cells were then counted using a hemocytometer with trypan blue staining. Cell viability was calculated based on the proportion of cells without trypan blue staining compared to the total number of cells. DMSO treatment was used as a control.

We previously demonstrated that 30 μM tetrandrine induced HCC cell apoptosis [29]. In contrast, 5 μM tetrandrine was sufficient to induce autophagy of liver cancer cells [30]. To determine whether tetrandrine would affect cell viability at the dose necessary for triggering autophagy, we treated MDA-MB-231, MCF-7, Hela and HFF cells with 10 μM tetrandrine for 24 hours and then assayed cell viability. We used rapamycin, a well-known inhibitor of the PI3K-mTOR pathway and autophagy inducer, as a control. As shown in Figure 1, 10 μM tetrandrine and 50nM rapamycin had almost no effect on cell survival in both nontumorigenic cell line HFF and tumor cell lines MDA-MB-231, MCF-7, Hela. Therefore, these results suggested that this low concentration of tetrandrine was nontoxic to cells.

### Tetrandrine potently induces autophagy in a variety of cell lines

**Figure 2:**
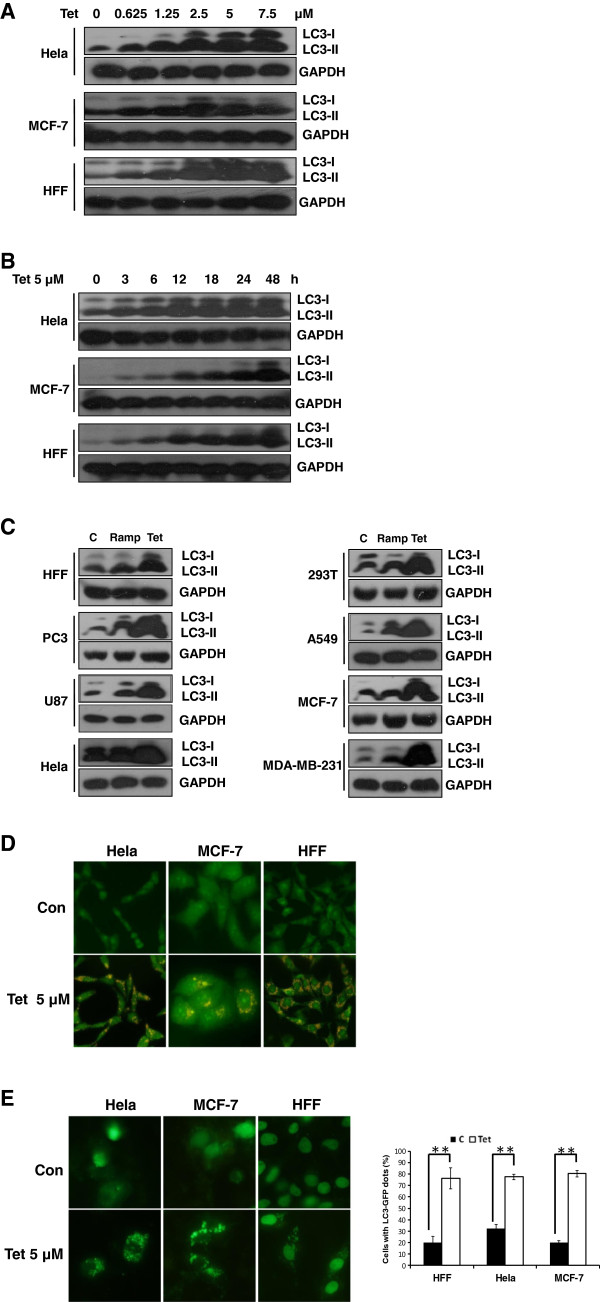
**Tetrandrine potently induces autophagy in a variety of cell lines.** Western blot analysis of autophagy-related LC3 proteins in Hela, MCF-7 and HFF cells after treatment with various concentrations **(A)** and gradient times **(B)** as indicated. **(C)** Cells described in the Materials and Methods section were treated with rapamycin or tetrandrine alone for 24 hours. Autophagy-related LC3 proteins were analyzed by western blot to assess autophagy induction. **(D)** Treatment with 5 μM tetrandrine induces autophagy in Hela, MCF-7 and HFF cells as analyzed by the acridine orange staining assays described in the Materials and Methods section. AO fluorescence was observed with a fluorescence microscope. **(E)** Cells with GFP-LC3 dots accumulation exhibited autophagy after tetrandrine treatment and were viewed by confocal microscopy, Error bars represent ± SD, **p < 0.01.

Although we had recently reported that tetrandrine induces HCC autophagy, it is still unclear whether tetrandrine can induce autophagy in other cells, such as cancer cells and nontumorigenic cells. We treated MCF-7, Hela and HFF cells with increasing concentrations of tetrandrine for 24 hours and then examined the expression of LC3-II, a membrane bound form of LC3 and an established marker of cell autophagy, via western blot analysis. As shown in Figure 2A, LC3-II protein level gradually increased in a dose-dependent manner with tetrandrine treatment. Similarly, tetrandrine induced autophagy of cells in a time-dependent pattern (Figure 2B). The above results indicated that treating cells with 2.5 μM tetrandrine for 12 hours can effectively induce autophagy. Further examination showed that tetrandrine also potently triggers autophagy in other cells, including PC3, U87, MDA-MB-231, A549 and HEK293. Moreover, treating cells with 2.5 μM tetrandrine and 50 nM rapamycin for 12 hours shown tetrandrine is a much stronger autophagy inducer than rapamycin (Figure 2C), and staining Hela, MCF-7 and HFF cells with acridine orange resulted in the formation of numerous acidic autophagolysosome vacuoles (AVOs) (Figure 2D). The formation of punctate spots with GFP-LC3 fusion protein is a well-characterized marker for visualizing autophagosomes. Here, we also observed the characteristic punctate fluorescent pattern of LC3-GFP when Hela, MCF-7 and HFF cells were transfected with the LC3-GFP plasmid and then treated with tetrandrine for 24 hours (Figure 2E). Collectively, these results suggest that tetrandrine is a potent autophagy inducer in a variety of cell lines.

### 3-methyladenine (3-MA) or chloroquine (CQ) blocked tetrandrine-induced autophagy

**Figure 3:**
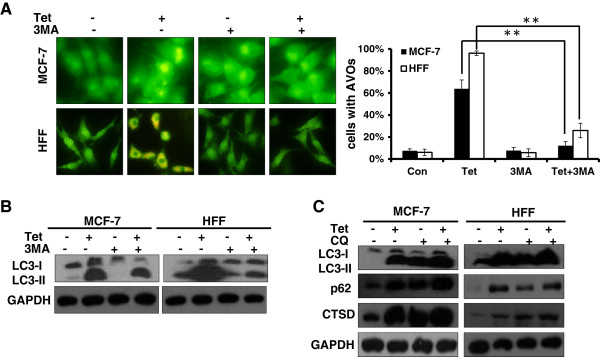
**3-methyladenine (3-MA) or chloroquine (CQ) blocked tetrandrine-induced autophagy. (A)** Autophagy analysis in MCF-7 and HFF cells treated with tetrandrine by acridine orange staining assays after a 1-hour pretreatment with 1.5 mM 3-MA. Error bars represent ± SD, **p < 0.01. **(B)** Western blot analysis of LC3-II protein levels before treatment or in the absence of 1.5 mM 3-MA in MCF-7 and HFF cells. **(C)** Western blot analysis of LC3-II, p62 and CTSD protein levels treated with tetrandrine for 24 hours after a 1-hour pretreatment with 15 mM CQ.

To further confirm our findings, we pretreated cells with 3-methyladenine (3-MA), a pan-PI3K inhibitor, for 1 hour before treatment with tetrandrine. The results showed that 3-MA partially blocked the formation of autophagolysosome vacuoles and inhibited the LC3-II levels that tetrandrine induced in MCF-7 and HFF cells by compare between tetrandrine add 3-MA group and tetrandrine alone group (Figure 3A and B). Chloroquine (CQ) is an inhibitor of autophagy flux that prevents autophagosome-lysosome fusion and lysosomal protein degradation by raising the lysosomal pH in the latter phase. As shown in Figure 3C, we found that chloroquine maintains the LC3-II, p62 and Cathepsin D (CTSD) protein levels and prevents their degradation by the lysosome compare to tetrandrine treated only, providing further validation that the tetrandrine-treated cells underwent autophagy.

### Tetrandrine triggered the induction of mitophagy

**Figure 4:**
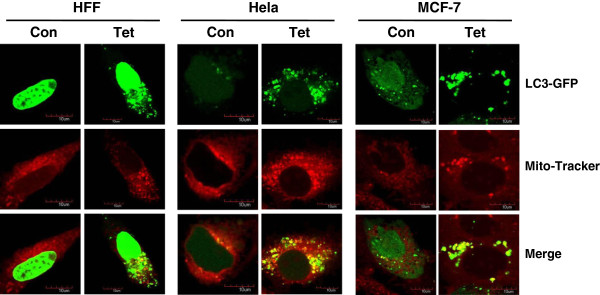
**Tetrandrine triggered induction of mitophagy.** Mitophagy was detected in GFP-LC3 expressing HFF, Hela and MCF-7 cells treated with 5 μM tetrandrine for 24 h by labeling with the MitoTracker Red dye and observing under a confocal microscope.

During autophagy, damaged organelles, such as mitochondria, can be engulfed by the double-membraned autophagosome before fusion with lysosomes, resulting in mitochondrial autophagy (mitophagy) [32]. To determine whether tetrandrine-induced autophagy involves mitophagy, we stained GFP-LC3-overexpressing Hela, MCF-7 and HFF cells with the MitoTracker Red dye and observed them by confocal microscopy. The results demonstrated that, when cells were treated with tetrandrine, autophagy been induced, then green punctae of GFP-LC3 and red light points of MitoTracker co-localized, indicating that cells underwent mitophagy (Figure 4). These findings suggested that tetrandrine triggered the induction of mitophagy.

### Intracellular reactive oxygen species (ROS) are essential in tetrandrine-induced autophagy

**Figure 5:**
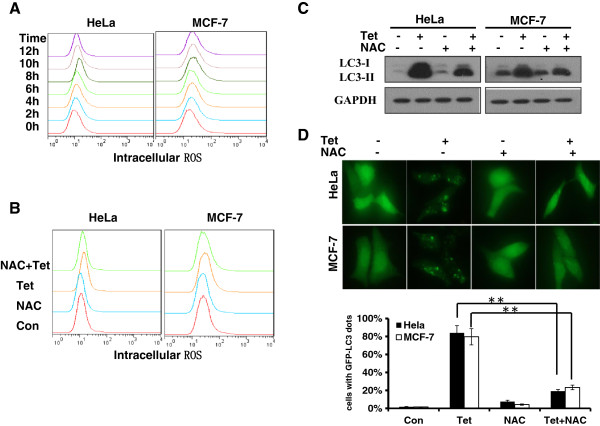
**ROS are essential in tetrandrine-induced autophagy. (A)** Intracellular ROS accumulation levels measured by flow cytometry after tetradrine treatment for the indicated times in Hela and MCF-7 cells. **(B)** Hela and MCF-7 cells were pretreated with 15 mM NAC for 1 hour and then with tetrandrine for 24 hours. Intracellular ROS levels were measured by flow cytometry. Autophagy detected by western blot analysis of LC3 levels **(C)** and GFP-LC3 dots accumulation assay **(D)** in cells pretreated with NAC, ** p < 0.01.

Cellular accumulation of ROS plays a vital role in the stimulation of autophagy under conditions of nutrient deficiency and chemotherapeutic agent treatment. The acute burst of ROS in mitochondria often leads to mitochondrial depolarization that then induces mitophagy. We next investigated whether tetrandrine-induced autophagy was associated with the production of ROS in Hela and MCF-7 cells. As shown as in Figure 5A and B, tetrandrine promoted a significant increase in intracellular ROS in a treatment time-dependent manner, and this increase was completely blocked by pretreatment with the reactive oxygen species scavenger N-acetyl- L-cysteine (NAC). Moreover, the ROS scavenger NAC could almost completely eliminate the ability of tetrandrine to induce autophagy, decreased the LC3-II levels and blocked the formation of green punctae of GFP-LC3 (Figure 5C and D), which suggests that the activation of intracellular ROS plays an essential role in tetrandrine-induced autophagy.

## Discussion

Exposure to cancer treatments (e.g., chemotherapy, radiotherapy, hormone therapy and targeted therapy), subjects cells to stress, which often induces cell autophagy [33, 34]. For cancer cells, autophagy serves a dual role, with both tumor-suppressing and tumor-promoting effects, by regulating cellular homeostasis [35]. Autophagy can become cytotoxic and lead to cell death if the stress imposed is too severe or prolonged [36, 37]. Therefore, cell autophagy agonists can potentially enhance the efficacy of cancer therapy and be used clinically in cancer treatment. In the present study, we demonstrated that tetrandrine is a broad-spectrum potent autophagy agonist exhibiting a stronger ability to induce cell autophagy than rapamycin that it can potently induce autophagy in a variety of cell lines, including cancerous cells and nontumorigenic cells, but the role underlie the effects on autophagy we still unknown. We previously reported that low doses of tetrandrine show good synergistic antitumor effects in combination with other chemotherapeutic agents, but has no cytotoxicity on normal cells [31]. Combined with our previous reports of the antitumor effects of tetrandrine, we speculated that tetrandrine may be a promising clinical cancer chemotherapeutic agent when used either alone or in combination with other drugs.

In response to chemotherapeutic drugs or radiation, sensitive cancer cells will eventually undergo different forms of death, including apoptosis, autophagic cell death, necrosis and senescence [36, 38]. Some researchers demonstrated that autophagy is often a prelude to many other forms of death. Different functions of autophagy occur in response to external stress [39]. Multiple regulatory genes have been reported to switch cells from cytoprotective to cytostatic autophagy in various cancer cell lines [40, 41]. Here, our studies showed that a low dose of tetrandrine did not affect cell proliferation and survival. However, at high concentrations, tetrandrine induces cancer cells apoptosis. Moreover, tetrandrine-induced autophagy is associated with the activation intracellular reactive oxygen species in a variety of cancer cells. It is worth mention that we used both effective methods to analyze autophagy including acid lysosome-autophagosome detection and quantification by acridine orange staining assays and LC3-II protein level analysis by western blot. Since acridine orange staining assays by fluorescent microscopy is less sensitive than western blot protein detection, the lysosome-autophagosome quantitative measurement showed slightly different from LC3-II protein level is normal under the premise of the same result.

Almost all types of anticancer agents, such as DNA damaging agents, antimetabolites, death receptor agonists, hormonal agents, antiangiogenic agents, proteasome inhibitors, histone deacetylase inhibitors, and some kinase inhibitors, have been shown to affect cell autophagy [33, 34]. Although the molecular mechanisms of autophagy are complex and numerous reports have reported conflicting roles for autophagy in cancer therapy, most researchers believe that autophagy might be a potential therapeutic target in cancer treatment [9, 10, 42]. In this sense, potent autophagy agonists or inhibitors with minimal toxicity are promising candidates for developing effective anticancer drugs [12, 13]. Tetrandrine is a traditional Chinese medicine that has been broadly used for thousands of years in China, making it suitable for development into a cancer therapy agent [43]. In addition, we believe that tetrandrine may act as an autophagy agonist in many systems.

## Conclusions

In summary, we present tetrandrine as a potent cell autophagy agonist for many types of cancer cells. It may have a wide range of applications in the fields of antitumor therapy and basic scientific research.

## Material and methods

### Chemical reagents and antibodies

Tetrandrine was purchased from Shanghai Ronghe Medical, Inc. (Shanghai, China) and dissolved in DMSO for use. DCFH-DA was obtained from Invitrogen (Carlsbad, CA). 3-Methyladenine (3-MA) and N-acetyl-L-cysteine were purchased from Sigma (St. Louis, MO). Acridine orange (AO), GAPDH antibody and HRP-conjugated secondary antibodies (goat anti-rabbit and goat anti-mouse) were purchased from Beyotime (Nantong, China). The antibody against microtubule-associated protein 1 light chain 3 (LC3) was purchased from Sigma (St. Louis, MO). The p62 antibody was obtained from Cell Signaling Technologies (Beverly, MA), and cathepsin D (CTSD) was acquired from Proteintech Group, Inc. (Chicago, IL).

### Cell lines and cell culture

The non-small-cell carcinoma cell line A549 and human prostate cancer cell line PC3 were cultured in complete1640 RPMI medium. The breast cancer cell lines MCF-7 and MDA-MB-231, glioma cell line U87, cervical cancer cell line Hela and immortalized nonmalignant cell line 293 T were cultured in Dulbecco’s modified Eagle’s medium (DMEM), and human foreskin fibroblast HFF were cultured in α-MEM medium (Gibco BRL, Grand Island, NY, USA); these media were supplemented with 10% fetal bovine serum (FBS, Hyclone), 1% penicillin and 1% streptomycin. Cells were cultured at 37°C in a humidified atmosphere of 95% air and 5% CO2. Cell culture dishes and plates were obtained from Wuxi NEST Biotechnology (Co., Ltd).

### Cell viability analysis

For cell viability assays, cells were observed using the trypan blue dye-exclusion assay. Cells were plated on 24-well plates and incubated with rapamycin or tetradrine for 24 h before being counted using a hemocytometer with trypan blue staining. After cells were harvested, cells were rinsed with PBS and then resuspended in 1 ml of PBS. A 10 μl aliquot of cell suspension was incubated with 10 μl 0.4% trypan blue solution for 5 minutes at room temperature. Viable and nonviable cells based on absence and presence of intracellular trypan blue dye, respectively. Percentages were counted by hemacytometer [44].

### Western blot analysis

After cells were harvested and lysed in 1% SDS on ice, cell lysates were immediately heated at 95°C for 15–20 minutes and then centrifuged at 12,000 × g for 10 minutes. The supernatant was collected, and the protein concentration was determined by the Pierce BCA Protein Assay Kit (Thermo Scientific). Equivalent amounts of protein (20 μg) from each sample were loaded and run on SDS-PAGE gels (Amresco), and then transferred to PVDF membranes (Millipore). After blocking the membranes with 5% non-fat milk (Bio-Rad) in Tris-buffered saline with 0.1% Tween-20 (TBST) at room temperature for 1 hour, the membranes were incubated with specific primary antibodies at 4°C overnight, washed with TBST three times (10 minutes each time), and incubated with HRP-conjugated secondary antibodies for 1 hour at room temperature. After washing with TBST, the immunoblots were visualized by chemiluminescence using a HRP substrate (Millipore). GAPDH was probed to ensure equal protein loading.

### Acridine orange staining assays

After tetradrine treatment, cells stained with acridine orange for acid lysosome-autophagosome were detected and quantified by fluorescent microscopy. In this assay, the intensity of the red fluorescence is proportional to the degree of acidity. Cells were collected, and the cells were resuspended in PBS and stained with AO (10 μg/ml) for 15 min at room temperature [45]. After washing with PBS, cells resuspended in 0.4 mL PBS, and the fluorescence of AO was viewed under a fluorescent microscope (Leica Microsystems GmbH). AVOs were accumulated in acidic spaces and fluoresces bright red punctuate staining dots in cytoplasm.

### Measurement of ROS accumulation

The intracellular ROS levels were detected using the DCFH-DA probe (Sigma) by flow cytometry. Dichloroflu orescein (DCFH), which has served as the workhorse for the redox biology community, detects multiple types of reactive small molecules [46]. Briefly, cells were harvested after treatment and washed twice with PBS, incubated with DCFH-DA (1 μM) in serum-free 1640 at 37°C in a 5% CO2 incubator for 20 minutes, washed twice with PBS and analyzed by flow cytometry. The data were processed using the FlowJo software (Tree Star, San Carlos, CA, USA).

### Transient transfection and autophagy detection

Cells were seeded on coverslips in 12-well plates. After 12 hours of growth, cells were transiently transfected with the pEGFP-LC3 plasmid according to the protocol. Twenty-four hours later, the cells were treated with tetrandrine. After treatment for 12 hours, the cells were viewed under a fluorescent microscope (Olympus BX51). The percentage of cells with more than five GFP-LC3 dots, which were considered to be autophagic, was quantified [47].

### Mitophagy detection

To detect mitophagy in cells [48], cells transiently transfected with the pEGFP-LC3 plasmid were treated with tetrandrine for 12 hours. Mito-tracker Red Dye was then added into the cell culture and incubated at 37°C for half an hour. Cells were then observed with a confocal fluorescent microscope.

### Statistical analysis

Results were expressed as the mean ± standard deviation (SD), and all statistical analyses were performed using Student’s t- test (two-tailed, unpaired). A P-value of 0.05 or less was considered significant.
